# Clinically informed machine learning elucidates the shape of hospice racial disparities within hospitals

**DOI:** 10.1038/s41746-023-00925-5

**Published:** 2023-10-12

**Authors:** Inas S. Khayal, A. James O’Malley, Amber E. Barnato

**Affiliations:** 1 The Dartmouth Institute for Health Policy and Clinical Practice, Geisel School of Medicine at Dartmouth, Lebanon, NH 03756 USA; 2 Biomedical Data Science, Geisel School of Medicine at Dartmouth, Lebanon, NH 03756 USA; 3 Department of Computer Science, Dartmouth College, Hanover, NH 03755 USA; 4 Cancer Population Sciences Program, Norris Cotton Cancer Center, Lebanon, NH 03756 USA; 5 Department of Mathematics, Dartmouth College, Hanover, NH 03755 USA; 6 Department of Medicine, Dartmouth-Hitchcock Medical Center, Lebanon, NH 03756 USA

**Keywords:** Palliative care, Quality of life, Health services

## Abstract

Racial disparities in hospice care are well documented for patients with cancer, but the existence, direction, and extent of disparity findings are contradictory across the literature. Current methods to identify racial disparities aggregate data to produce single-value quality measures that exclude important patient quality elements and, consequently, lack information to identify actionable equity improvement insights. Our goal was to develop an explainable machine learning approach that elucidates healthcare disparities and provides more actionable quality improvement information. We infused clinical information with engineering systems modeling and data science to develop a time-by-utilization profile per patient group at each hospital using US Medicare hospice utilization data for a cohort of patients with advanced (poor-prognosis) cancer that died April-December 2016. We calculated the difference between group profiles for people of color and white people to identify racial disparity signatures. Using machine learning, we clustered racial disparity signatures across hospitals and compared these clusters to classic quality measures and hospital characteristics. With 45,125 patients across 362 hospitals, we identified 7 clusters; 4 clusters (*n* = 190 hospitals) showed more hospice utilization by people of color than white people, 2 clusters (*n* = 106) showed more hospice utilization by white people than people of color, and 1 cluster (*n* = 66) showed no difference. Within-hospital racial disparity behaviors cannot be predicted from quality measures, showing how the true shape of disparities can be distorted through the lens of quality measures. This approach elucidates the shape of hospice racial disparities algorithmically from the same data used to calculate quality measures.

## Introduction

Racial disparities in hospice care are well documented for patients with cancer, but the findings are contradictory^1^. At the end of life, several study findings have revealed racial disparities in hospice care between white people and people of color^2–6^, where people of color utilized disproportionately less hospice care. On the other hand, other studies have concluded that no differences exist in hospice utilization^7–9^. With regard to hospice length of stay, Ngo-Metzger et al. have shown no significant difference in length of hospice stays between racial or ethnic subgroups of Asian Americans and Pacific Islanders (AAPIs), which include Chinese, Filipino, Japanese Americans, and Hawaiian/Pacific Islanders^10^. Ngo-Metzger et al. have also shown that all AAPI subgroups were less likely than white people to enroll in hospice^10^. And yet, despite lower enrollment, Park et al. found that length of hospice care is actually longer for people of color than white people^11^. For late hospice use, Miesfeldt et al. found higher Black versus non-Black late hospice use^12^, while others found no difference in late hospice utilization within 3 days^2^ or for hospice stays greater than 3 days^13^. Still, other studies disagree with the prior findings and indicate that rates of late and early hospice initiation were similar across racial/ethnic groups^2^. Consequently, not only is identifying a disparity in hospice use at the end-of-life important but to account for the value of hospice to patients, the timing of initiation (e.g., early, late) and length of stay are important considerations.

The data to assess racial disparities research is heterogeneous and ranges from a single organization’s electronic medical records to national-level claims across hospitals. Different data types allow for different analyses; namely, a within or across-hospital analysis. At first glance, reports of racial/ethnic healthcare disparities are likely to be attributed entirely to unequal treatment within a hospital. However, it is critical to note that researchers have posited that these disparities, based on *across-hospital* analyses, arise primarily because of where people live geographically because people of color tend to live in parts of the country with a disproportionate share of low-quality hospitals^14–19^. On the other hand, *within-hospital* healthcare disparities arise primarily because of unequal treatment within a hospital. Attempts at within-hospital analyses using claims have been approached by calculating quality measures for each patient group and performing a pairwise comparison–effectively requiring across-hospital quality and hospital factors data for the calculation. Effectively, the analysis of two numbers within a hospital is insufficient to understand if a disparity exists. Furthermore, these numbers provide limited to no insights about why, when, where, or what behaviors affect this disparity. This analysis is again problematic because it is driven by the same place-based disparities described above. This problematic approach may explain why single hospital systems with the ability to analyze local racial disparities may not have found similar findings^7,8^.

While the method of analysis may seem trivial or too detailed, different analytic decisions and specifications may directly impact the policy agenda on addressing disparities. Findings that racial disparities are predominately driven by region of residence than by ethnicity at hospitals serving a high-fraction of people of color delivering poorer quality of care has led to a policy agenda that focuses on specific hospitals. And yet, our national agenda should focus on eliminating all racial disparities everywhere. Indeed, equitable care is one of the six domains of healthcare quality set forth by the Institute of Medicine^20^, and one of the last remaining domains to garner the attention it deserves. Therefore, a national agenda to address equity requires innovative and efficient quantitative methods to identify quality and within-hospital disparities for all hospitals, in which a deeper understanding of quality, and of the timing and magnitude of racial disparities are elucidated.

In this paper, we describe an innovative explainable machine learning approach to elucidate within-hospital racial disparities from administrative claims data. We incorporate clinical information and leverage a systems engineering approach to describe the behavior of hospice utilization by racial groups as a longitudinal signature. In addition, we employ machine learning to classify these signatures into groups with similar underlying disparity patterns and show how disparities can be distorted through the lens of quality measures. Finally, elucidating heterogeneity in within-hospital disparities may help explain the mixed findings in the literature and highlight the importance of developing a public health strategy focused on reducing local disparities with bespoke solutions.

## Results

### Cohort

We attributed 126,434 Medicare beneficiary decedents to 2174 US hospitals. Of these decedents, 22,020 (17.4%) were people of color. For this paper, we included only hospitals that had beneficiaries that included at least 11 people of color and 11 white people, for a total of 45,125 beneficiaries that died at 362 hospitals, of which 11,625 (20.48%) were people of color. The percentage of people of color attributed to each hospital ranged from 6.2% to 82% with a median and mean of 27% and 29.7%, respectively. The 362 hospitals included 18 National Cancer Institute–Designated Cancer Centers (NCI) that are not National Comprehensive Cancer Network Centers (NCCN), 22 NCCN centers, 55 academic medical centers, and 267 community hospitals. Most hospitals were in urban areas, with 356 hospitals located in a metropolitan area core, 5 hospitals located in a micropolitan area core and 1 hospital located in a micropolitan high commuting area, as defined by the rural-urban commuting area (RUCA) primary codes^21^, which delineate sub-county components of rural and urban areas.

### Racial disparity signatures and their classification

Smoothed hospice racial disparity signatures for all hospitals are available in a public, open access repository in the Dartmouth Dataverse at 10.21989/D9/9DLP65. Although disparity signature values can theoretically range from −100% to 100% for the last 6 months before death, we found the data ranged from −42.69% to 31.77% with a median and mean of 0% and −0.39%.

**Fig. 1:**
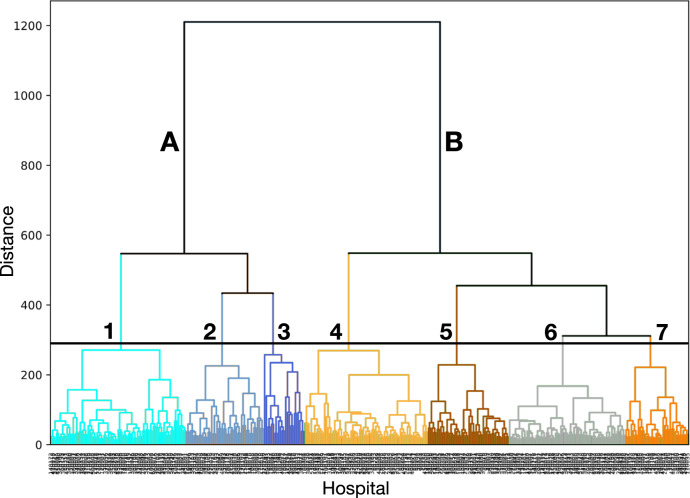
A dendrogram diagram showing the hierarchical relationships for all hospitals based on the agglomerative hierarchical analysis distance. We annotated the identified 7 clusters (1–7) within 2 classes (**A**, **B**).

**Fig. 2:**
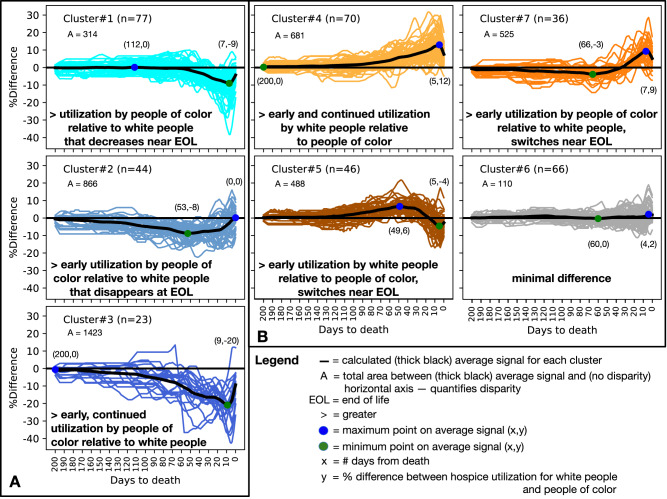
Each hospital disparity signature is visualized into its labeled cluster subplot. Clusters 1-3 are associated with class **A** and all show higher hospice utilization by people of color relative to white people. Clusters 4–7 are associated with class **B**. Clusters 4 and 7 end with higher hospice utilization by white people than people of color. Clusters 2, 5, and 6 end around no disparities between groups but show very different patterns prior to death with a very different overall disparity calculated as the total area between the signature and the horizontal axis (corresponding to no disparity). We also include a narrative description of the racial disparity for each cluster. The thick black line is the calculated average signal for each cluster. The value A quantifies disparity as the total area between the black average signal and the horizontal (no disparity) line. EOL is an abbreviation for end of life. Blue circles represent the maximum point on the thick black average signal (x,y), and the green circle represents the minimum point on the thick black average signal. In the (x,y) point notation, x represents the number of days from death and y represents the percent difference between hospice utilization for white people and people of color.

We identified 7 clusters in the agglomerative hierarchical clustering. We visualized the clusters in Fig. 1 as a dendrogram “tree diagram”, where the y-axis tree depth corresponds to the distances between clusters and the x-axis represents a vertical line for each hospital. The first 3 clusters are closely related through a single branch A and the last 4 clusters are related through a single branch B. We labeled each hospital based on the cluster it connected to in the dendrogram. In Fig. 2, we visualized the hospital disparity signatures for all hospitals assigned to each of the 7 clusters in a separate subplot. We calculated the average disparity signature for each cluster and drew it as a thick black line. We annotated each (black) average disparity signature with the maximum and minimum points to highlight the differences between the signatures. We also visualized the absolute area between the average signature and the horizontal axis (corresponding to no disparity) and a narrative description of each cluster in Fig. 2. Specifically, we defined the shape of disparity as the absolute area between the average signature and the no disparity horizontal axis. In other words, the shape of the disparity signature represents the direction, timing, duration, and intensity of racial and ethnic disparity in hospice utilization for the last 6 months of life.

Clusters 1, 2, and 3 predominantly showed higher hospice utilization by people of color relative to white people. The differences between these 3 clusters highlighted the timing of when the difference occurs (*x*-axis) and the extent of the difference (*y*-axis). Cluster 4 and 7 both ended in the last month with higher hospice utilization by white people relative to people of color, but for cluster 4, the difference appeared earlier and began closer to 3 months before death, whereas cluster 7 actually showed the opposite, slightly higher hospice utilization by people of color relative to white people 2–4 months prior to death. Cluster 5, first showed higher utilization by white people relative to people of color, similar to cluster 4, until about 1 month prior to death, when hospice utilization by people of color increased and overtook utilization by white people with a peak at 5 days before death and ends on the day of death with almost no disparity in hospice utilization. Finally, cluster 6 showed an average signal that is mostly flat around 0 suggesting a minimal difference in hospice utilization over time within these hospitals.

### Hospice disparity signatures and hospital factors

Analyzing hospice quality measures for white people and people of color per hospital, we found a significant Spearman correlation (*r* = 0.5828, *p* = 3.94*10^−33^) and a weak R^2^ = 0.3397. A pairwise comparison using the Wilcoxon signed-rank test showed hospice quality measures were significantly different (*p* = 1.12*10^−7^) between white people and people of color. Hospitals showed higher (*n* = 226), lower (*n* = 135), and equal (*n* = 1) hospice values for white people than for people of color. Calculating the percent difference between hospice quality measures for white people and people of color, we found no significant correlation between the hospice disparity signature and the hospice quality measure for all patients at each hospital (*p* = 0.8301), or between the hospice disparity signature and the percent of people of color attributed to each hospital (*p* = 0.4677). We found a weak significant Spearman correlation between the hospice quality measure for all patients and the percent of patients served in a hospital that are people of color (i.e., percentage of people of color attributed to each hospital) (*r* = −0.1746, *p* = 0.0008), and a very weak R^2^ = 0.0335.

**Fig. 3:**
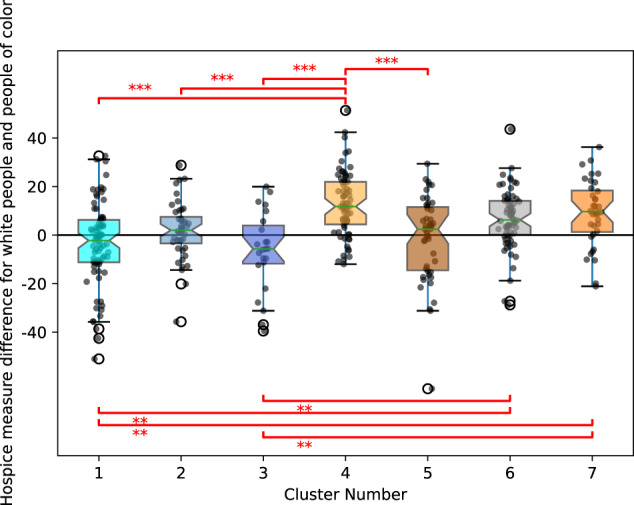
Box plot values of hospice quality measure differences between white people and people of color for hospitals in each of the 7 clusters. The box extends from the Q1 to Q3 quartile values of the data, with a line at the median (Q2), and Tukey-style whiskers extend to a maximum of 1.5 × IQR (IQR = Q3-Q1) beyond the box. We also visualize the actual data points. Significant differences (based on the Conover-Iman test) are annotated in red, where ***p < =0.0005 and **p < =0.005. In other words, at the time of death, hospitals in clusters 1, 2, 3, and 5 tended to end with more hospice utilization by people of color relative to white people, relative to hospitals in cluster 4. Also, hospitals in clusters 1 and 3 had more hospice utilization by people of color than white people near death than hospitals in clusters 6 and 7.

Next, we compared the different hospital clusters with hospital characteristics. For continuous variables, the Kruskall–Wallis test showed no significant difference in the hospice quality measure for all patients at each hospital (*p* = 0.0606) or the percentage of people of color attributed to each hospital (*p* = 0.8408) between the 7 clusters. On the other hand, there was a significant difference between the hospice quality measure difference between white people and people of color (*p* < 7.36 × 10^−11^) and the total number of patients treated at each hospital (*p* = 0.0086) between the 7 clusters. We visualized a box plot of the hospice quality measure differences between white people and people of color for each of the 7 clusters in Fig. 3. Specifically, based on the Conover-Iman test, the hospice quality measure differences between white people and people of color had median differences that were significantly different in cluster 4 than clusters 1 (*p* = 1.5 × 10^−9^), 2 (*p* = 0.0005), 3 (*p* = 2.4 × 10^−6^), and 5 (*p* = 6.8 × 10^−5^), and median differences that were significantly different in clusters 6 and 7 than clusters 1 (*p* = 0.0013 and *p* = 0.0024, respectively) and 3 (*p* = 0.0016 and *p* = 0.0029, respectively). These results were not surprising. They point to the fact that clustering hospitals based on the full disparity signature would lead to different groups than if only the very end of the disparity signature at time of death (*t* = 0) was used. The machine learning clustering placed clusters 1, 2, and 3 together because they clearly showed a total negative disparity area with more hospice utilization by people of color than white people (class A), and clusters 4–7 together because they showed an element of a positive disparity area with more hospice utilization by white people than people of color (class B). In contrast, clustering based on the end of the signature at the time of death, hospitals in clusters 1, 2, 3, and 5 tended to end with more hospice utilization by people of color relative to white people than hospitals in cluster 4 and hospitals in clusters 1 and 3 have more hospice utilization by people of color than white people near death than hospitals in clusters 6 and 7. In addition, the number of patients treated at hospitals in cluster 6 tended to be larger than those in cluster 3 (*p* = 0.006).

**Fig. 4:**
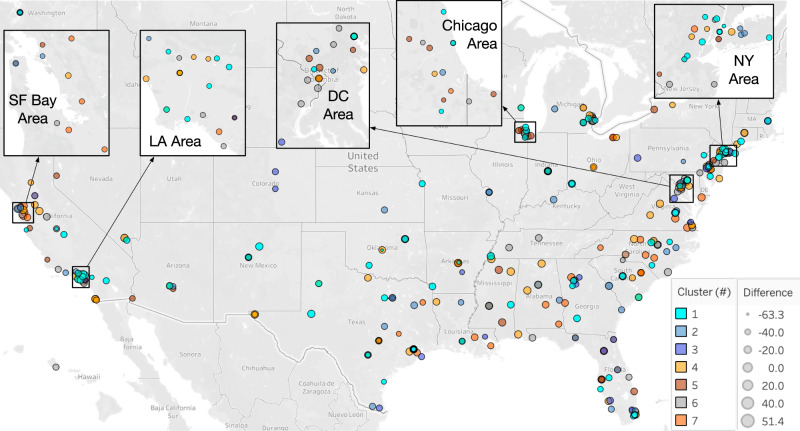
Each hospital is geolocated on the US map with a circular marker using Tableau (Academic License). Marker color represents the cluster number and size represents the hospice quality measure difference between people of color and white people. We zoomed into 5 areas to highlight the heterogeneity of clusters within very close geographic proximity areas in New York (NY), Chicago, District of Columbia (DC), Los Angeles (LA), and San Francisco (SF).

For nominal variables, the Pearson chi-squared test showed no significant difference in values between the 7 clusters for rurality (*p* = 0.8251), hospital type (*p* = 0.3879), city (*p* = 0.4560), or state (*p* = 0.0646). In Fig. 4, we visualized the geolocation of the 362 hospitals on a US map. We represented the marker size with the hospice quality measure difference between white people and people of color for each hospital, and the marker color with one of the 7 clusters. We zoomed into five regions to highlight the range of clusters within very close geographic proximity.

## Discussion

In this study, we developed a clinically-informed machine learning approach to explicitly elucidate within hospital racial and ethnic disparities. We used this approach with the same administrative claims data, typically used for generating quality measures, to generate disparity signatures that transparently show the shape of hospice disparity over time. Our findings suggest that heterogeneous hospice racial disparity signatures (1) elucidate disparity in hospice utilization over time, confirming the importance of the time dimension, (2) provide actionable timing and magnitude information, supporting decision-making for quality and equity improvement, and (3) provide interpretable disparity results even when hospitals improve over time, which otherwise would lead to poor machine learning performance and utility when changes in underlying patterns (concept drift) occurs.

Methodologically, our approach of clinically-informed machine learning (ML) produces interpretable and explainable results relative to conventional ML, which identifies “strong, but theory-free, associations in the data”^22^. In this analysis, we incorporated clinical constructs that take into account important patient health outcome information–time in hospice care. Our approach of explicitly incorporating time “informs” machine learning; an approach previously suggested to advance conventional ML with physical information^23,24^ or theory/knowledge^25^, to address the key limitation of black-box ML, where the lack of explainability can lead to low trust. Said differently, there is a growing need for explainable machine learning^26^, which can be achieved by infusing relevant domain knowledge into the machine learning process, for understandable, interpretable, and therefore transparent and trustworthy results from these approaches.

Hospice disparity signatures clearly show a variation in racial disparity over time before death; confirming the importance of the time-dimension. Consequently, achieving a difference of 0 in the last few days of life is not equitable or fair; the value of hospice is in the cumulative time a person receives hospice care (length of hospice stay^27^). Hospice disparity signatures are related to two categories of disparities, (1) statistical parity, “where each group receives an equal fraction of possible outcomes”–since our formulation of the difference between groups is prefaced on statistical parity between the groups across time– and (2) disparate impact: “a quantity that captures whether wildly different outcomes are observed in different groups”–which is exactly what the difference signature captures as the signal deviation from the horizontal-zero-line^28,29^. Given that value of care to the patient is associated with time in hospice care, it becomes clearer how an analysis incorporating time of when and how long care was delivered allows for a closer description of measurable quality to patients and their families. Ethnicity-based healthcare disparities within hospitals do not appear to vary geographically or correlate to any of our tested exogenous factors. Consequently, better health quality and equity comes from delving inward to understand internal care processes and factors, as suggested by the literature^30^. For example, palliative care consults have been shown to affect hospice use, demonstrating how local processes likely affect hospice use and length of stay^31–33^.

In addition to confirming the importance of an internal focus, hospice disparity signatures provide an analytical approach to analyzing within-hospital racial disparities that provide specific timing and magnitude differences that can serve as possible targets for quality and equity improvement. Findings from previous across-hospital analyses have led researchers to suggest policies that call for reducing healthcare disparities by targeting hospitals with poorer quality measures that disproportionately serve people of color^15^, rather than a focus on every hospital needing to improve racial inequity for all their patients. While healthcare equity is grounded in the belief that everyone has fair and just access to healthcare, individualized-hospital quality and equity improvement is only plausible when quality information can be used to guide improvement. While the incentives for learning health systems have evolved and become ripe with value-based care under accountable care organizations^34^, the science and informatics have been lacking with quality measures falling short on delivering the insights needed for learning or improvement^35,36^. To address this limitation, quality signatures provide longitudinal directional and magnitude information on an effect that can help guide exploration into EMR patient records to identify groups of patients with different hospice utilization such that questions surrounding hospice use and patient-level factors (e.g., patient preferences and factors–such as social determinants of health^37^), and system-level factors (e.g., care by specific providers or teams, and care processes) can be explored for racial inequity. Studies have shown the importance of both patient preferences^38–41^ and hospital factors^42–44^ on end-of-life care utilization. Furthermore, quality signatures can minimize perverse incentives to improve quality measures that lead to late referral to hospice that is harmful to caregivers and patients^45^. Therefore, this approach also reduces the misalignment between improving quality by focusing on quality measures versus quality of patient care, by transparently showing *when* hospice utilization occurs and the days of hospice utilization showing a disparity between racial groups.

Learning requires a cycle of continuous improvement using updated information. Johnson et al. have specifically called for future work to examine changes over time by ethnicity to better clarify whether use of these services has become more similar or differences have widened^33^. Conventional ML algorithms are not ideal in this case of change, since ML models fundamentally require the underlying associations to remain unchanged to continue to be valid^22^, which cannot be an accurate assumption if the goal is change. On the other hand, quality signatures are easily computed and directly reflect the timing and magnitude of change in racial disparity.

Our findings should be interpreted with a number of potential limitations in mind. We applied this method to hospitals with a large enough patient pool, 11 or more people of color and 11 or more white people. This approach leaves many hospitals with no ability to quantify disparity signatures, but this focuses on the importance of a strong enough signal to make claims of racial disparity. In addition, combining care patterns from people of different ethnic backgrounds who may have different experiences of bias in healthcare delivery is a limitation of this work. In future work, we will pool several years of data across centers to calculate Black-specific and hospice utilization rates. We used the decedent follow-back method, which assumes that studying individuals prior to their death is equivalent to studying care received by individuals who are dying^46^. In most practical situations, the two are unlikely to be equivalent as the distribution of observed and unobserved characteristics in people known to have died may be quite different from those with an elevated risk of dying in the near future (although the difference ought to lessen with the severity of the patients status among those considered incurable). To alleviate and address this distinction, future studies will apply a prospective (forward) method to understand the care received by individuals who are dying from advanced cancer. In addition, racial disparities only include Medicare patients seen at a hospital at some point in the last 6 months of their care, therefore, signatures only reflect racial disparities for the population that included a hospital visit. In this population, hospice referral at the end of life occurred through inpatient referral to hospice or through outpatient practice/oncology delivery system if the patient had a hospital visit in the last 6 months of life. The “decision” about hospice enrollment is likely a complex one across outpatient oncology (primary), consultants (palliative care, when involved), and inpatient providers (oncology consultant on service who talks to a primary oncologist or is the primary oncologist, palliative care, hospital medicine, ICU medicine, social work, etc.). Future studies, will not impose inclusion criteria of a hospital visit in the last 6 months of life, but may instead use other criteria to associate a patient with a hospital, such as an outpatient clinic relationship with a particular hospital. There is a potential that societal and systematic bias, such as racism, may have led to selection effects with fewer people of color living long enough (65+) to enter Medicare, which may lead to a healthier subset than the general population (the healthy survivor phenomenon), but it is also likely that these same biases may shift the healthy survivor effect in the opposite (negative) direction as well. Future research may address this using data from younger commercially or Medicaid-insured patients. Our analysis did not include a detailed set of endogenous and exogenous factors, such as those related to the patient, hospital, or society. However, in instances where such data is more available (e.g., within a hospital/EMR), it can be combined with information from signatures to provide a much more nuanced analysis of factors that affect these signatures. To address the two prior limitations, future work will develop hospital-facing tools to allow hospitals to produce racial disparity signatures applied to their own locally available data.

Racial disparities signatures identify within-hospital disparities from the same claims data source that has been used to provide across-hospital disparity insights, indicating a remarkable increase in efficiency. Future work will provide tools for hospitals to apply this approach to identify local within-hospital disparities. This approach can be extended and applied to other types of disparities beyond ethnicity, such as gender, socioeconomic, or rural, and for other diseases. This approach can also be used to incorporate hospice utilization signatures with other utilization signatures for palliative care, advance care planning, intensive care unit, emergency department, and others^47^, to leverage the rest of the patient’s utilization record and incorporate a holistic analysis of a patient’s care utilization.

## Methods

### Patient cohort and hospice quality measures

The patient cohort included Medicare fee-for-service beneficiaries with advanced (poor-prognosis) cancers, defined as cancers that carry a high risk of near-term death. We identified poor prognosis advanced cancers as metastatic cancers and primary cancers associated with high-risk mortality based on the methods of Iezzoni and colleagues^48^, which were adapted to the International Classification of Diseases, Tenth Revision, Clinical Modification (ICD- 10-CM) by ref. ^49^. The inclusion criteria included beneficiaries that: (1) died between April 1, 2016 and December 31, 2016, (2) are between the ages 66 and 99, (3) had at least one admission for cancer in the last 6 months of life, and (4) for whom there existed a complete 6-month look-back period between October 1, 2015 and March 31, 2016. The look-back period was used to identify when a patient utilized hospice care. We used the Medicare fee-for-service data from a retrospective study of decedents completed by ref. ^49^ More specifically, we used a 100% sample of Medicare fee-for-service beneficiaries drawn from 2015–2016 Centers for Medicare and Medicaid Services (CMS) files, including: (1) the Master Beneficiary Summary file, (2) the Medicare Provider Analysis and Review (MedPAR) file, (3) Physician/Supplier Carrier file, (4) the Outpatient file, (5) the Hospice file.

Each beneficiary was attributed to the hospital providing the preponderance of cancer care hospitalizations in the last 6 months of life, as previously defined by ref. ^49^ In addition, based on patient attributions to hospitals, we calculated the percentage of people of color attributed to each hospital. CMS claims include a modified beneficiary ethnicity code that takes the beneficiary ethnicity code that has historically been used by the Social Security Administration and applies an algorithm that enhances ethnicity designation based on first and last names to identify more beneficiaries that are Hispanic or Asian^50,51^. We used the previously calculated “Proportion Not Admitted To Hospice” quality measure (NQF #0215), as defined and endorsed by the National Quality Forum^52^, hereafter referred to as “hospice quality measure”. The hospice quality measure values are openly available in a replication data repository for this patient cohort^53^. A hospice quality measure was calculated for people of color and white people at each US hospital. In this study, we only included hospitals with at least 11 decedent white people and 11 decedent people of color, which we based on CMS suppression rules. Hospital characteristics included hospital type National Cancer Institute–Designated Cancer Centers (NCI), National Comprehensive Cancer Network Centers (NCCN), Academic Medical Centers (AMC), and Community Hospitals), city, state, percentage of people of color attributed to each hospital, rurality, and the number of patients with advanced cancer treated. We identified hospital rurality from publicly available 2010 Rural-Urban Commuting Area (RUCA) codes from the U.S. Department of Agriculture website^21^.

*The Dartmouth Health Institutional Review Board (Dartmouth Health Human Research Protection Program) approved this study and determined that this research is not human subjects research because the data is from decedents and not living humans (IRB STUDY02000656)*.

### Hospice quality signatures calculations

At a meta-theory level, this method is a gray-box hybrid modeling approach^54^ that used clinical information and engineering systems modeling to explicitly quantify disparity as group-based time-varying behavior of care utilization and used this disparity as the input data to an unsupervised machine learning model. We juxtapose this approach with the classic black-box approach that utilizes feature engineering to identify a model’s input data in a supervised or unsupervised machine learning model.

This section details the methodology to calculate hospice quality signatures based on a model-based systems engineering framework applied to healthcare delivery^55^ to describe the dynamic behavior of care utilization in healthcare systems^56,57^. Specifically, we first reconstructed each patient’s Medicare hospice utilization into a time-based vector showing which days the patient was either in hospice (value = 1) or not in hospice (value = 0) to produce a 6-month vector representing hospice utilization. Second, we created a higher-level behavior signal for a group by summing the signals for the individuals in the group and normalizing them by the number of people in the group, as a form of flexible hierarchical aggregation^57^ that results in a function that represents the percentage of the group utilizing hospice over time. Third, we explicitly calculated a difference signal that captures the difference in hospice utilization behavior over time.

**Fig. 5:**
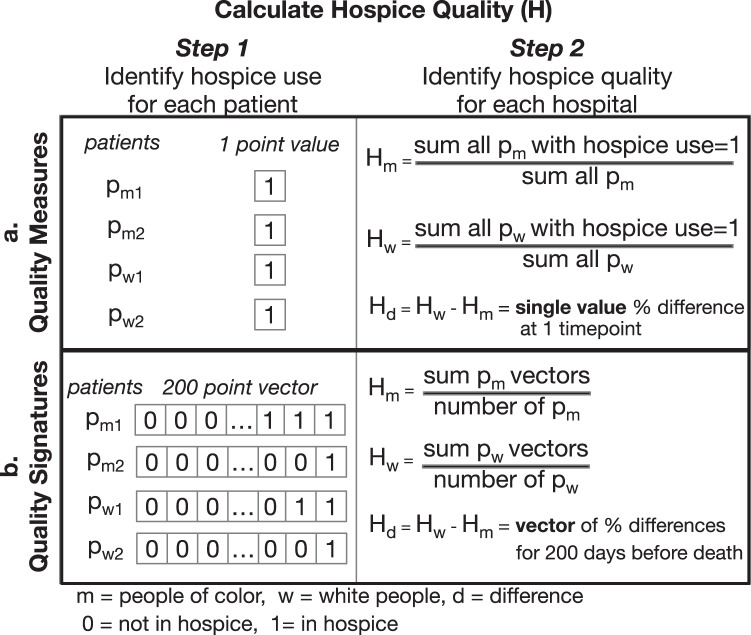
Hospice measures and signature calculations. **a** Quality Measures produce a single value for either day of death or a specific day before death. **b** Quality Signatures produce a vector of percent differences for the day of death, as well as for 199 prior days.

An overview of this methodology included a comparison of how claims are used for quality measure calculations, in Fig. 5. For each patient with advanced cancer, hospice claims were used to identify which days prior to death they were enrolled in hospice. This data was converted into a 200-day vector of values (i.e., signal), with 0 for days not in hospice and 1 for days in hospice. From a clinical perspective, patients that enter hospice tend to remain in hospice until death. Consequently, this generally creates a visual step-like function of 0’s until a specific day out from death when a patient enters hospice and tends to remain in hospice. For each hospital, patients were classified into racial groups of white people (Non-Hispanic white) or people of color (Black or African-American, Asian/Pacific Islander, Hispanic, American Indian/Alaska Native, Unknown, Other) using the beneficiary ethnicity code from the Master Beneficiary Summary file^58^. The signals for all patients belonging to a group were summed (i.e., time-dependent means or vector means) to generate a signal for people of color and another for white people for each hospital. The signals were then normalized by dividing the signal values by the number of patients in each group. Next, a straightforward difference was calculated by subtracting the signal for people of color from the signal for white people to produce the difference signal, which includes positive or negative values from −100 to 100. In the case when the daily value is an event indicator variable, the difference described here is a difference of percentages. Positive values represent the percentage of higher hospice utilization by white people relative to people of color, and negative values represent the percentage of higher hospice utilization by people of color relative to white people. Each hospital difference signal represents the disparity signature for that hospital, which in the simple case of binary indicators for hospice status on a given day corresponds to the average difference in the number of days in hospice spent by white people compared to people of color at that hospital.

To examine the variation of hospice disparity signatures across hospitals, we applied an unsupervised machine learning algorithm to group similar signatures into clusters. First, we smoothed each hospital hospice disparity signature by calculating the discrete, linear convolution of the original hospice disparity signature with a 10-day kernel window. Second, we applied a “bottom up” agglomerative hierarchical clustering algorithm, which starts with many small clusters and merges them together to create bigger clusters. Clusters are merged by calculating the distance between sets of observations, also called the linkage criterion. We tested several linkage criteria and chose the Ward’s minimum variance method^59^, based on the formation of dendrograms and visual inspection of their clusters. We chose the number of groups based on a combination of the Elbow method^60^, the formation of dendrograms, and visual inspection of their clusters.

### Statistical analysis

First, we analyzed the overall hospice quality measures and the specific hospice measures for white people and people of color for each hospital. We tested the quality measures for normality and appropriately applied non-parametric tests. We determined the Spearman correlation and calculated the R^2^ coefficient of determination. We then performed a pairwise comparison of quality measures for white people and people of color at each hospital using the Wilcoxon signed-rank test. Next, we explored the relationship between hospital clusters and hospital characteristics. We conducted a Kruskall–Wallis test for continuous variables. For significant *p* < 0.05 values, we followed this analysis with a Conover-Iman test and adjusted the *p*-value for multiple comparisons by applying a step-down method using Bonferroni adjustments. For nominal variables having sufficient (greater than 5) numerator and denominator counts, we conducted a one-sided Pearson Chi-Square test. We also plotted each hospital on a US map, with its size corresponding to the percent difference between the hospice quality measures for white people and people of color. We also color-coded each hospital based on the cluster it was assigned by the machine learning analysis. All analyses and visualizations were completed using Python 3.7 and Tableau 2021.3.13 Academic License.

### Reporting summary

Further information on research design is available in the Nature Research Reporting Summary linked to this article.

### Supplementary information

Reporting Summary

## Data Availability

The datasets generated and/or analyzed during the current study are available in the Dartmouth Dataverse repository, 10.21989/D9/9DLP65.

## Code availability

The underlying code for this study is not publicly available, but may be made available to qualified researchers on reasonable request from the corresponding author.
