# Supplementary material for: Clinically informed machine learning elucidates the shape of hospice racial disparities within hospitals
Source: NPJ Digit Med. 2023 Oct 12;6:190. doi: 10.1038/s41746-023-00925-5 (PMC10570342; doi:10.1038/s41746-023-00925-5)
Supplement: Supplementary file 1 — Reporting Summary [file 41746_2023_925_MOESM1_ESM.pdf]

nature portfolio

Corresponding author(s): Inas Khayal

Last updated by author(s): Jul 5, 2023

Reporting Summary

Nature Portfolio wishes to improve the reproducibility of the work that we publish. This form provides structure for consistency and transparency in reporting. For further information on Nature Portfolio policies, see our Editorial Policies and the Editorial Policy Checklist.

Statistics

For all statistical analyses, confirm that the following items are present in the figure legend, table legend, main text, or Methods section.

n/a	Confirmed	
☐	☒	The exact sample size (*n*) for each experimental group/condition, given as a discrete number and unit of measurement
☐	☒	A statement on whether measurements were taken from distinct samples or whether the same sample was measured repeatedly
☐	☒	The statistical test(s) used AND whether they are one- or two-sided<br>*Only common tests should be described solely by name; describe more complex techniques in the Methods section.*
☒	☐	A description of all covariates tested
☐	☒	A description of any assumptions or corrections, such as tests of normality and adjustment for multiple comparisons
☒	☐	A full description of the statistical parameters including central tendency (e.g. means) or other basic estimates (e.g. regression coefficient) AND variation (e.g. standard deviation) or associated estimates of uncertainty (e.g. confidence intervals)
☐	☒	For null hypothesis testing, the test statistic (e.g. *F*, *t*, *r*) with confidence intervals, effect sizes, degrees of freedom and *P* value noted<br>*Give P values as exact values whenever suitable.*
☒	☐	For Bayesian analysis, information on the choice of priors and Markov chain Monte Carlo settings
☒	☐	For hierarchical and complex designs, identification of the appropriate level for tests and full reporting of outcomes
☐	☒	Estimates of effect sizes (e.g. Cohen's *d*, Pearson's *r*), indicating how they were calculated

Our web collection on statistics for biologists contains articles on many of the points above.

Software and code

Policy information about availability of computer code

Data collection	Openly available Python software and packages (pandas, numpy) were used to calculate the disparity signatures as detailed in the manuscript.
Data analysis	Openly available Python software and packages (sklearn, scipy) were used to run the agglomerative hierarchical clustering algorithm.

For manuscripts utilizing custom algorithms or software that are central to the research but not yet described in published literature, software must be made available to editors and reviewers. We strongly encourage code deposition in a community repository (e.g. GitHub). See the Nature Portfolio guidelines for submitting code & software for further information.

Data

Policy information about availability of data

All manuscripts must include a data availability statement. This statement should provide the following information, where applicable:

- Accession codes, unique identifiers, or web links for publicly available datasets
- A description of any restrictions on data availability
- For clinical datasets or third party data, please ensure that the statement adheres to our policy

The datasets generated and/or analyzed during the current study are available in the Dartmouth Dataverse repository, https://doi.org/10.21989/D9/9DLP65.

Research involving human participants, their data, or biological material

Policy information about studies with human participants or human data. See also policy information about sex, gender (identity/presentation), and sexual orientation and race, ethnicity and racism.

Reporting on sex and gender	This research focused on race and ethnicity and not on sex or gender.
Reporting on race, ethnicity, or other socially relevant groupings	We constructed a group called people of color to represent all races and ethnicity that are not white non-hispanic. This is because the population size is small and would have limited the number of hospitals we could analyze.
Population characteristics	The population is for patients with poor-prognosis advanced cancers. We identified poor prognosis advanced cancers as metastatic cancers and primary cancers associated with high risk mortality based on the methods of Iezzoni and colleagues, which were adapted to the International Classification of Diseases, Tenth Revision, Clinical Modification (ICD-10-CM) by Wasp et al.
Recruitment	No patients were recruited. We used Medicare beneficiary decedent data.
Ethics oversight	The Dartmouth Health Institutional Review Board (Dartmouth Health Human Research Protection Program)

Note that full information on the approval of the study protocol must also be provided in the manuscript.

Field-specific reporting

Please select the one below that is the best fit for your research. If you are not sure, read the appropriate sections before making your selection.

☐ Life sciences ☒ Behavioural & social sciences ☐ Ecological, evolutionary & environmental sciences

For a reference copy of the document with all sections, see nature.com/documents/nr-reporting-summary-flat.pdf

Behavioural & social sciences study design

All studies must disclose on these points even when the disclosure is negative.

Study description	This study was a quantitative study
Research sample	We identified hospice utilization for deceased Medicare Beneficiaries with poor-prognosis advanced cancers. Hospice care is considered high quality care for patients with cancer and especially poor-prognosis advanced cancer.
Sampling strategy	We identified all patients with poor-prognosis advanced cancers that died in 2016 that provides a complete 6-month look back period. Therefore we sampled all patients.
Data collection	Administrative data collected directly through the practice of clinical care.
Timing	We identified patients that died between April-December 2016
Data exclusions	No exclusions.
Non-participation	Not applicable.
Randomization	No randomization.

Reporting for specific materials, systems and methods

We require information from authors about some types of materials, experimental systems and methods used in many studies. Here, indicate whether each material, system or method listed is relevant to your study. If you are not sure if a list item applies to your research, read the appropriate section before selecting a response.

Materials & experimental systems

n/a	Involved in the study	
☒	☐	Antibodies
☒	☐	Eukaryotic cell lines
☒	☐	Palaeontology and archaeology
☒	☐	Animals and other organisms
☐	☒	Clinical data
☒	☐	Dual use research of concern
☒	☐	Plants

Methods

n/a	Involved in the study	
☒	☐	ChIP-seq
☒	☐	Flow cytometry
☒	☐	MRI-based neuroimaging

Clinical data

Policy information about clinical studies

All manuscripts should comply with the ICMJE guidelines for publication of clinical research and a completed CONSORT checklist must be included with all submissions.

Clinical trial registration	Not applicable
Study protocol	Not applicable
Data collection	Data provided directly from the Centers for Medicare and Medicaid Services through a data use agreement.
Outcomes	No outcome measures were assessed. Utilization of hospice care was our data.
